# Occupational noise exposure in manufacturing industries in Malaysia

**DOI:** 10.1186/1471-2458-14-S1-O17

**Published:** 2014-01-29

**Authors:** Noraita Tahir, Syed Mohamed Aljunid, Jamal Hisham Hashim

**Affiliations:** 1 United Nations University International Institute for Global Health (UNU-IIGH), 56000 Cheras, Kuala Lumpur, Malaysia

## Background

Manufacturing sector accounts for 18.1% of the total employed persons in Malaysia, which is known for the utilisation of noisy machinery to establish output. Even though it is compulsory for employers to notify any hearing loss cases to the authority annually, there is still scarcity of occupational hearing loss cases. Therefore, this study aimed to estimate the total number of workers exposed to occupational noise among workers in manufacturing industries in Malaysia.

## Materials and methods

A field survey was carried out at 26 selected industries in five states Johor, Selangor, Pulau Pinang, Terengganu and Pahang that recorded highest noise induced hearing loss (NIHL) cases in 2011. Safety and Health Practitioners in each industry were interviewed and elicited information on the industrial classification, occupational noise management and level of exposure to workers. Noise exposures were categorised from noise exposure monitoring report. The percentage of high risk workers in each class was derived to estimate the number of exposed workers based on the Economic Census.

## Results

Levels of occupational noise exposure among industries were 28% for 91-140 dBA and 72% for 86-90 dBA; which classified that all workforces were at high risk. Occupational noise-exposed workers were observed to be highest in the metal industry (2091) followed by textile (631) and food (439). The percentage of workers exposed ranged from 13.6% to as high as 68.9% in each industry. In addition 103,673 (39%) from total employment of 267,964 were estimated to be workers exposed to high risk noise. Although, employers provide awareness programme and hearing protection device but none of the industries implemented attenuation of it. A few industries showed inconsistency in annual audiometric test.

## Conclusions

This study indicates that the majority of workforce in manufacturing industry was exposed to high risk occupational noise and effective hearing conservation programme needs to be implemented for preventive measure.

